# 3D printing of sacrificial templates into hierarchical porous materials

**DOI:** 10.1038/s41598-018-36789-z

**Published:** 2019-01-23

**Authors:** Lauriane Alison, Stefano Menasce, Florian Bouville, Elena Tervoort, Iacopo Mattich, Alessandro Ofner, André R. Studart

**Affiliations:** Complex Materials, Department of Materials, ETH Zürich, Vladimir-Prelog-Weg 5, 8093, Zürich, Switzerland

## Abstract

Hierarchical porous materials are widespread in nature and find an increasing number of applications as catalytic supports, biological scaffolds and lightweight structures. Recent advances in additive manufacturing and 3D printing technologies have enabled the digital fabrication of porous materials in the form of lattices, cellular structures and foams across multiple length scales. However, current approaches do not allow for the fast manufacturing of bulk porous materials featuring pore sizes that span broadly from macroscopic dimensions down to the nanoscale. Here, ink formulations are designed and investigated to enable 3D printing of hierarchical materials displaying porosity at the nano-, micro- and macroscales. Pores are generated upon removal of nanodroplets and microscale templates present in the initial ink. Using particles to stabilize the droplet templates is key to obtain Pickering nanoemulsions that can be 3D printed through direct ink writing. The combination of such self-assembled templates with the spatial control offered by the printing process allows for the digital manufacturing of hierarchical materials exhibiting thus far inaccessible multiscale porosity and complex geometries.

## Introduction

Highly porous materials combine low density with mechanical, thermal and electrical properties that can be deliberately tuned according to the aimed application and functionalities^1^. Applications range broadly from lightweight structures^2^ and thermal management^3^ to catalysis^4,5^, filtration^6^ and chemical sensing^5^. Porous structures have also been exploited for optics^5^, energy storage^7^, tissue engineering and drug delivery^8^. In many of these applications, hierarchical structures with pores of distinct sizes are desired to achieve a proper balance between conflicting properties. Examples where such balance is achieved are found in hierarchical structures that combine high permeability with high accessible surface area or high mechanical properties with low weight. Such hierarchical porous structures have been produced using a variety of synthetic approaches. However, porous architectures obtained thus far do not reach the level of complexity observed in porous materials created by living organisms in Nature. Exquisite porous architectures found in Nature include the skeleton of marine sponges^9,10^, the vasculature system of plants^10^, the trabecular structure of bone tissue^9,10^, and the internal structure of wood^9^ and bamboo^2^. To further improve the level of control over the architecture of synthetic porous materials, it is crucial to develop novel routes for the manufacturing of hierarchical porous structures^11^.

Porous materials have been synthetically produced using replica techniques, emulsion templating approaches, direct foaming, capillary suspensions, and additive manufacturing^12–15^. Emulsion templating and direct foaming are particularly interesting because the oil droplets and air bubbles of emulsions and foams can be easily tuned to generate materials spanning a wide range of porosities and pore sizes. However, the thermodynamically unstable nature of emulsions and foams make these pore templates susceptible to coarsening mechanisms that prevent accurate control over pore sizes. To address this issue, particle-stabilized foams and emulsions with enhanced resistance against coarsening and coalescence have been used as templates for the fabrication of tunable porous materials^16^. The stabilization of oil droplets via the adsorption of particles at the oil-water interface lead to so-called Pickering emulsions. The use of these soft templates as inks in extrusion-based 3D printing^13,17^ proved to be an effective means to generate hierarchical porous materials through additive manufacturing^18^. In this approach, the nozzle path followed during 3D printing is used to define the pore sizes at larger length scales, whereas the porosity at finer length scales is determined by the size of templating droplets and bubbles. This approach has been utilized to create a wide variety of hierarchical cellular materials^18–21^. Typically, the porous architectures obtained exhibit pores ranging from millimetres down to a few micrometers in size.

Despite the promising prospects of utilizing emulsions and foams as inks for additive manufacturing, producing porous hierarchical structures with smaller pore sizes would increase further the potential of this technology in applications requiring high surface area, optical transparency and high mechanical properties. To fulfill this demand, emulsions with droplet sizes in the submicron range could be used as inks. Although Pickering emulsions typically exhibit larger droplet sizes, a few examples have been reported of submicron particle-stabilized droplets. This has been possible by synergistically combining particles and surfactants during emulsion stabilization^22,23^. In this approach, surfactants are used to quickly diffuse and adsorb at the oil-water interface. This facilitates the break-up of droplets into submicron sizes during emulsification due to the reduced tension of the fluid interface. Moreover, it prevents coalescence of the submicron droplets at short timescales. Subsequent adsorption of particles at the interface ensures the stabilization of droplets against coarsening at longer timescales. Emulsions produced through this route can be further processed by spray drying^24,25^, but the obtained structures have been so far limited to materials in granular or colloidal form of relevance for pharmaceutical applications^26,27^.

Here, we 3D print inks that consist of nanoemulsions and other microtemplates to produce complex-shaped hierarchical materials with controlled pores ranging from hundreds of nanometers to millimetres in size. The pore size of the resulting porous materials can be easily tuned through the selection of the printing path and the size of the pore templating building blocks. Submicron pores are generated from particle-stabilized nanoemulsions, whereas larger droplets or sacrificial polymer particles are used to create pores in a size range varying from 10 to 100 µm. Finally, the macroscopic complex shape and the large-scale cellular architecture of the hierarchical porous material is determined by the 3D printing process. To illustrate our approach, we first describe the processing route used to produce the nanoemulsions. This is followed by a thorough investigation of the structure of the porous materials obtained when such soft nano-templates are combined with larger emulsion droplets or polymer particles. Finally, we demonstrate how these pore templating building blocks can be used as ink constituents to 3D print porous materials with complex shape and internal porous structure of relevance for several applications but that is not easily accessible with other manufacturing technologies.

## Results and Discussion

**Figure 1:**
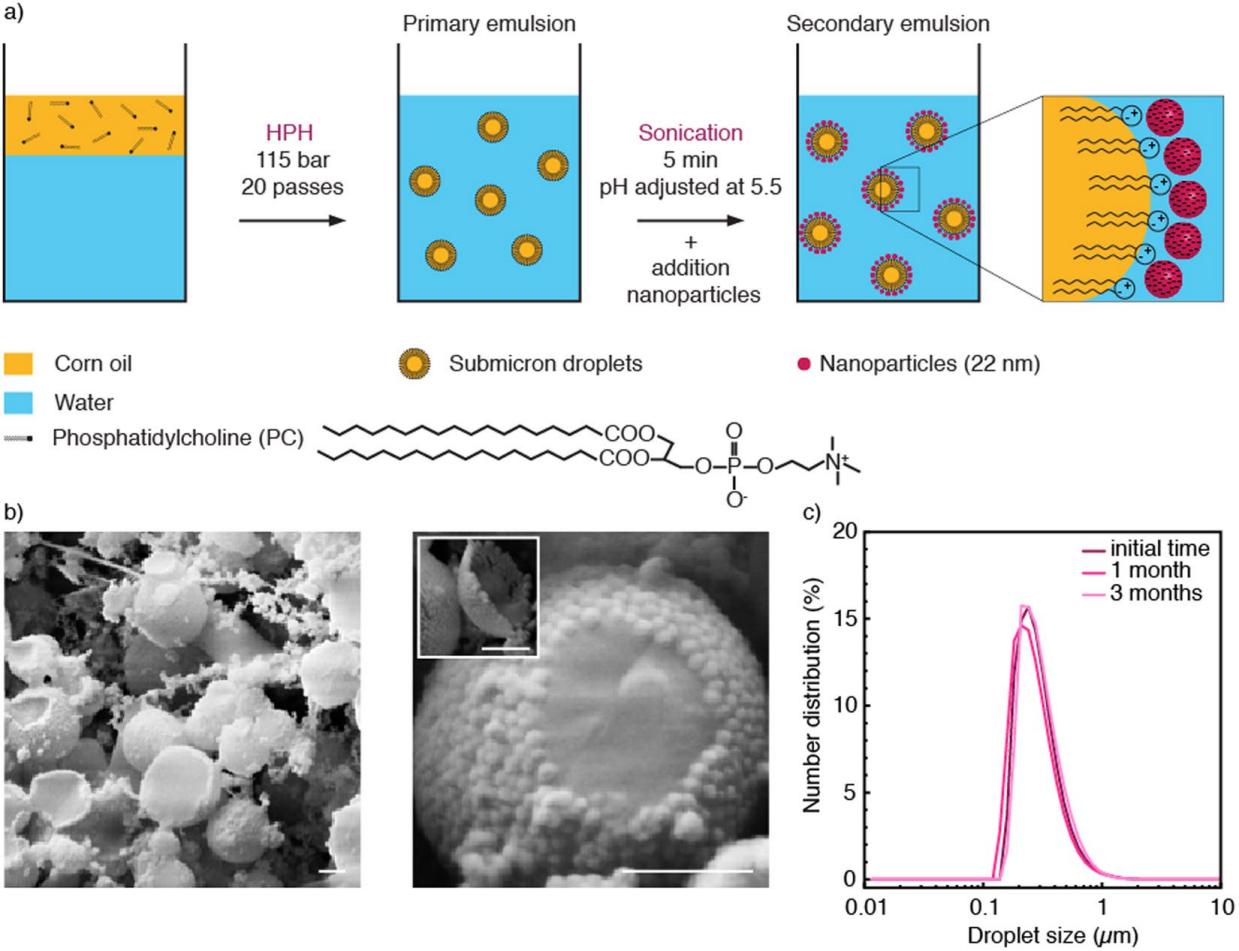
(**a**) Formation of nanoemulsions via a synergistic stabilization mechanism involving a zwitterionic surfactant phosphatidylcholine (PC) and silica nanoparticles. HPH stands for high-pressure homogenizer. (**b**) Cryo-SEM images of the resulting particle-stabilized nanodroplets. The inset shows a silica particle monolayer that detached from the droplet surface during sample preparation. Scale bars in B: 200 nm. (**c**) Evolution of the droplet size distribution over time for nanoemulsions stabilized by 7 wt_O/W_% particles and 1 wt_oil_% surfactant.

Following concepts outlined in earlier works^22,23,28^, stable nanodroplets are formed in a two-step emulsification process through the synergistic action of hydrophilic nanoparticles and an oil-soluble zwitterionic surfactant. We illustrate this process using phosphatidylcholine (PC) and silica nanoparticles as zwitterionic surfactant and hydrophilic nanoparticles, respectively (Fig. 1a). The zwitterionic surfactant effectively stabilizes micron-sized droplets of corn oil suspended in water during a first emulsification step. In a second emulsification step, the nanoparticles are introduced in the continuous aqueous phase of the primary-formed emulsions and the resulting mixture is subjected to ultrasonication. The high energy introduced through sonication lead to further break-up of the oil phase, decreasing the droplet size to the nanoscale. During this process, excess PC molecules present in the oil phase are expected to adsorb rapidly at the oil-water interface, reducing its interfacial tension and facilitating droplet break-up. This approach circumvents the slow diffusion rates and adsorption dynamics of particles at liquid interfaces^29^, thus enabling the stabilization of smaller droplet sizes. Observations of the emulsion microstructure by cryo-scanning electron microscopy (cryo-SEM) show that the silica nanoparticles adsorb as a dense layer around the oil nanodroplets (Fig. 1b). Such dense layer exhibits a viscoelastic behavior that prevents coarsening and coalescence of the nanodroplets for long time periods, as evidenced by interfacial rheology measurements of the oil-water interface (Fig. S1a). Because the primary-formed droplets carry predominantly negative electric charges at the processing pH, electrostatic forces alone are not enough to explain the adsorption of negatively-charged nanoparticles to the droplet surface. Instead, adsorption may result from non-electrostatic forces and other interactions possibly involving the positive charges of the zwitterionic surfactant present at the oil-water interface (Fig. S1b). By contrast, no viscoelastic film is formed when only particles or PC are used in the formulation, which results in larger and less stable droplets after emulsification (Fig. S2). As demonstrated in previous studies^30–32^, the interfacial adsorption of particles is an effective mechanism to stabilize emulsions against coalescence and Ostwald ripening. Indeed, dynamic light scattering experiments show that the interfacial viscoelastic film enables the stabilization of nano-sized droplets for time periods as long as 3 months (Fig. 1c).

**Figure 2:**
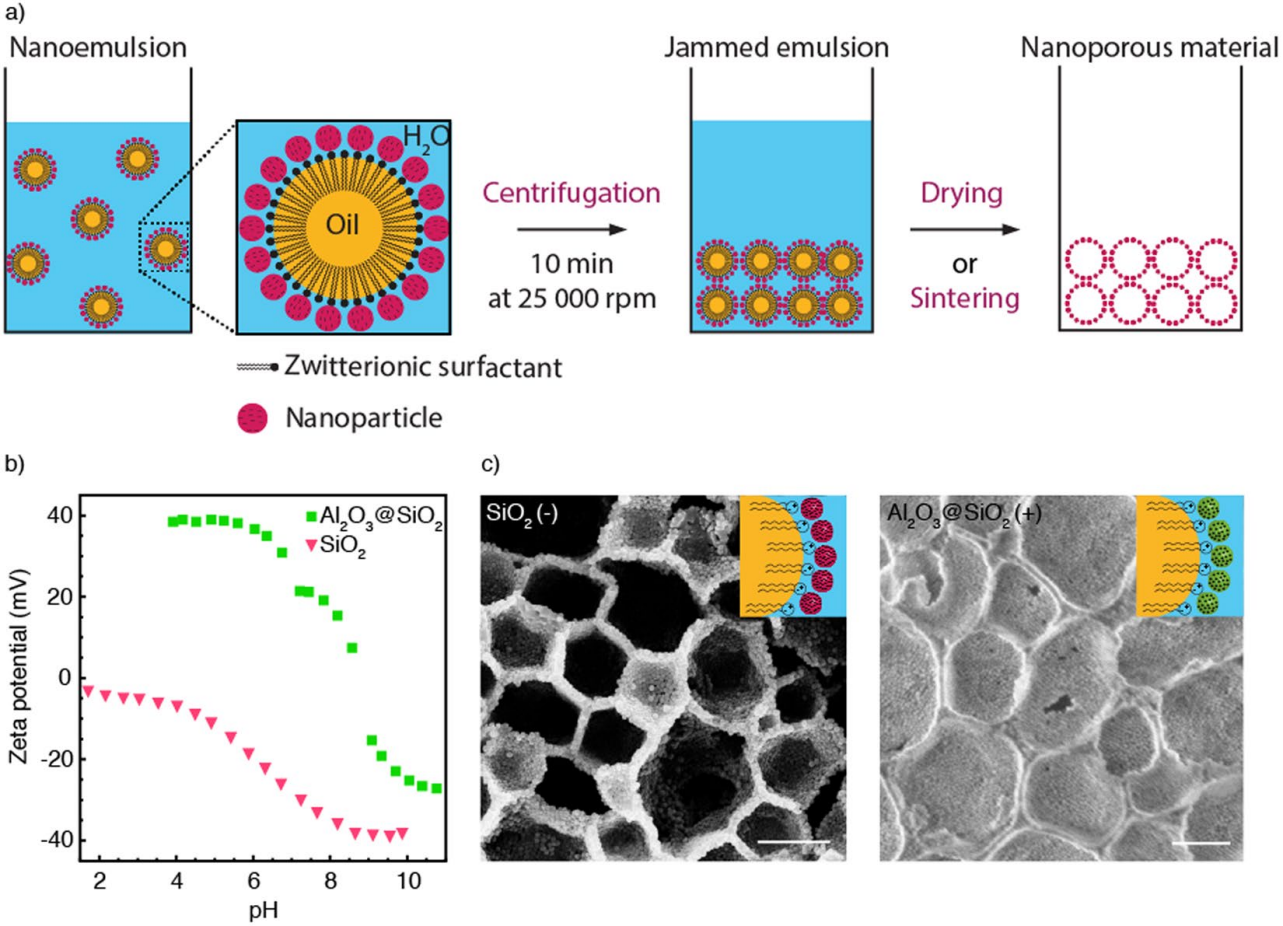
(**a**) Schematics illustrating the preparation of nanoporous structures from particle-stabilized nanoemulsions. (**b**) Zeta potential measurements of bare silica and alumina-coated silica particles used for emulsion stabilization. (**c**) SEM images of the porous structures obtained after ultracentrifugation and sintering of nanodroplets stabilized using negatively (left) and positively charged (right) nanoparticles and prepared with corn oil (non-volatile). Scale bars: c, 400 nm.

Nanodroplets formed through this processing route are stable enough to be concentrated by ultracentrifugation and thus form a dense jammed template that can be directly converted into a nanoporous structure upon drying or sintering (Fig. 2a) depending on the oil volatility. The porosity of the structure is controlled by the concentration of oil droplets in the jammed nanoemulsion, which was estimated to be higher than 70 vol%. To show that our processing route is applicable to a wide range of materials, we fabricated nanoporous structures using nanoparticles of distinct surface chemistries (Fig. 2b,c). In addition to bare silica, alumina-coated silica nanoparticles were used as model building blocks for this purpose. By using coated particles, we demonstrate the versatility of the method while keeping the same particle size as the reference silica powder. The versatility arises from the zwitterionic nature of the surfactant, which is able to electrostatically attract both positively and negatively-charged hydrophilic particles during the second emulsification process (Fig. S1a).

Bare silica (Ludox TM50) and alumina-coated silica particles (Ludox CL-P) show indeed negative and positive net surface charges, respectively, at the processing pH of 5.5. This was confirmed by zeta potential measurements of such nanoparticles in water as a function of pH (Fig. 2b). Using PC as zwitterionic surfactant, stable nanoemulsions were also successfully prepared using the alumina-coated silica nanoparticles. Sintering of the centrifuged emulsions led to porous structures with either bare silica or alumina-coated silica nanoparticles as the main building blocks in the pore walls (Fig. 2c). Alternatively to sintering, chemical strategies can also be utilized to consolidate the structure if temperature-sensitive constituents need to be preserved, as discussed later^20^. To confirm the flexibility of the method, porous structures were also successfully produced using smaller 7 nm silica particles and bare alumina particles as building blocks (Fig. S3).

The size of the nanopores obtained after drying and sintering directly reflects the size of the precursor nanodroplet templates. Taking the system with bare silica nanoparticles as an example, we observe that nanoemulsions with droplet size in the range of 150–1000 nm (Fig. S2) lead to porous structures with pore sizes varying from 100 to 900 nm after drying and sintering. Because the nanoparticles form a dense layer on the surface of the precursor droplets, closed nanopores are often obtained after drying and sintering. However, open pores can also form if the emulsions are slightly destabilized during processing to generate droplet surfaces that are only partially covered by particles. For the emulsions investigated in this work, we found that such slight destabilization is possible by replacing corn oil by decane as the dispersed phase (Fig. S4). The ability to tune the process to generate either open or close porosity after sintering enables tailoring of the porous structure according to the properties required by the aimed application.

In addition to nanoporous structures, the nanoemulsions can also be combined with micron-sized sacrificial templates to generate porous architectures featuring a second level of porosity. Two exemplary types of sacrificial templates are used here to create micron-sized pores: oil droplets and polymer particles. The choice of the second sacrificial template affects the possible consolidation step and the resulting porous structures. If micron-sized oil droplets are used, the wet emulsion template can be conveniently consolidated by simple evaporation of the liquid phases followed by chemical cross-linking of the remaining constituents. By contrast, polymer particle templates require a sintering step for consolidation if long dissolution of the solid polymer phase is to be avoided. Besides such distinct processing pathways, oil droplets are more prone to deformation and distortion during the drying and consolidation steps. This contrasts with the undeformable nature of polymer particle templates.

**Figure 3:**
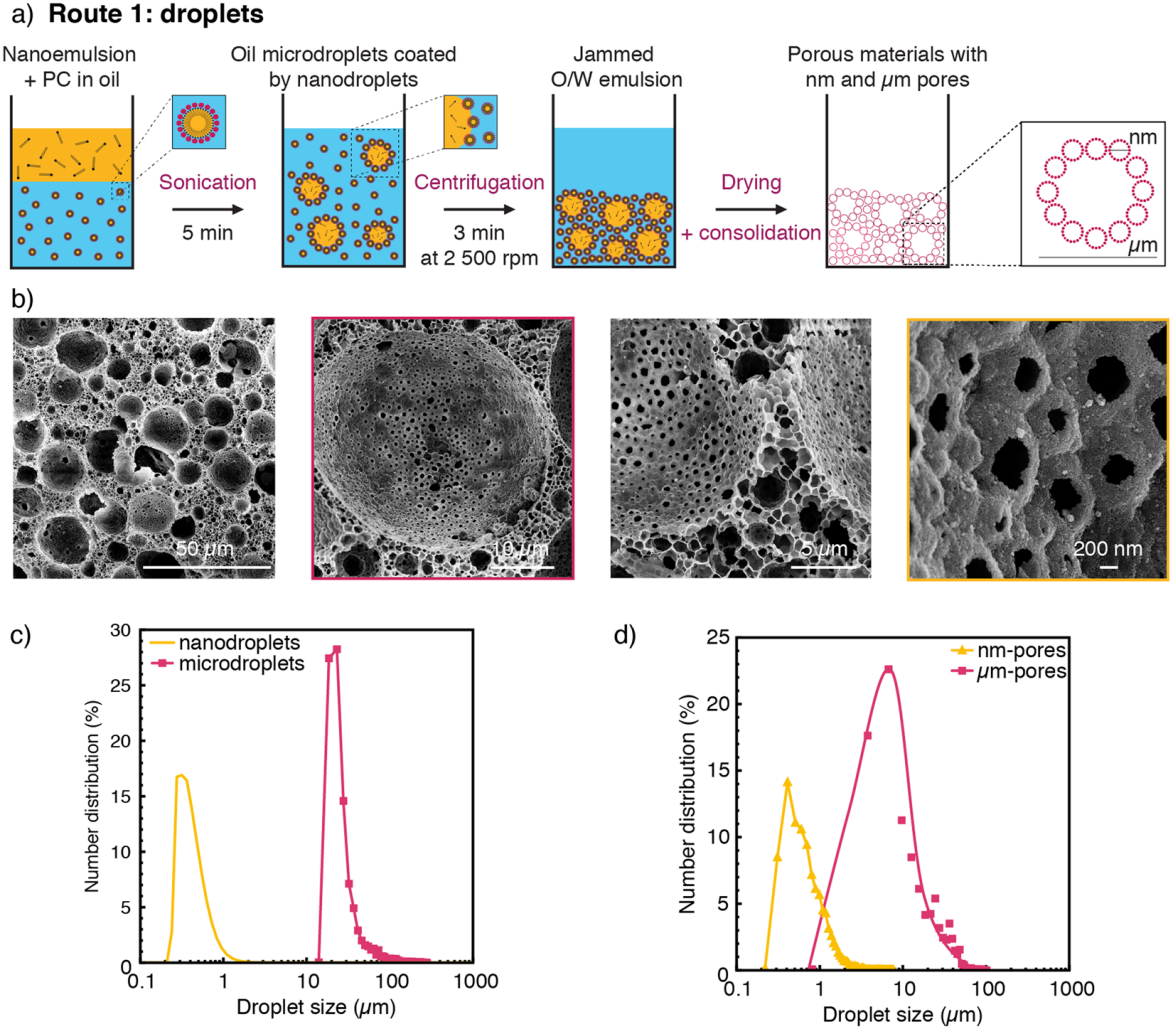
(**a**) Schematics of the processing route used to fabricate hierarchical porous materials from nanoemulsions and microdroplets (Route 1) (**b**) SEM images of the porous structure obtained after drying of the soft precursors. (**c**,**d**) Size distributions of the µm-templates and the nm-sized droplets (**c**) before and (**d**) after drying.

We illustrate the different possible pathways towards two-level hierarchical porosity by using polycaprolactone (PCL) particles or decane droplets as the micron-sized sacrificial templates (Fig. 3). When oil droplets are used as second soft template (Route 1), an additional emulsification step is employed to create large oil droplets from the previously prepared nanoemulsions (Fig. 3a). Confocal microscopy imaging confirmed the adsorption of the nanodroplets on the surface of the larger oil droplets (Fig. S5), which is important to prevent the phase separation of the two droplet sizes during centrifugation. The size of the larger droplets can be typically varied between 15 and 250 µm, depending on the intensity and duration of the emulsification process. Simple drying of the liquids from the multiscale emulsion at 25 °C results in a porous structure featuring pore sizes at the nanometer and micrometer scales (Fig. 3b). This porous structure can be consolidated and strengthened at room temperature by crosslinking of chitosan molecules previously added to the emulsion continuous phase. To illustrate this possibility, 5 wt% chitosan was incorporated prior ultracentrifugation into the aqueous phase of the nanoemulsion to enable cross-linking with 2.5 wt% glutaraldehyde (Fig. S6). This room-temperature consolidation process is particularly suitable for the preparation of polymer-based bio-scaffolds, since it prevents the thermal degradation of the organic matter during heat treatment. Analysis of the pore size distribution of the porous structures obtained after drying shows the two expected families of pores (Fig. 3c,d). The size distribution of the smaller pores agrees well with the droplet size distribution of the original nanoemulsion template. This suggests that the small dimensions of the nanodroplets prevent their distortion during the drying process. In contrast to the size fidelity observed at small scales, the size distribution of larger pores was found to be broader as compared to the size distribution of the templating droplets. Such an effect is probably a result of the shrinkage that takes place during drying and the associated capillary forces that develop within the structure during removal of the liquid phases. Despite their small sizes, the nanopores generated on the walls of macropores (Fig. 3b) were sufficient to generate an open porosity level as high as 90% in the final sintered structure. Open porosity with larger windows between macropores can be generated by reducing the stability of the templating emulsion using for example lower concentrations of particle stabilizers or surface modifiers^33^.

**Figure 4:**
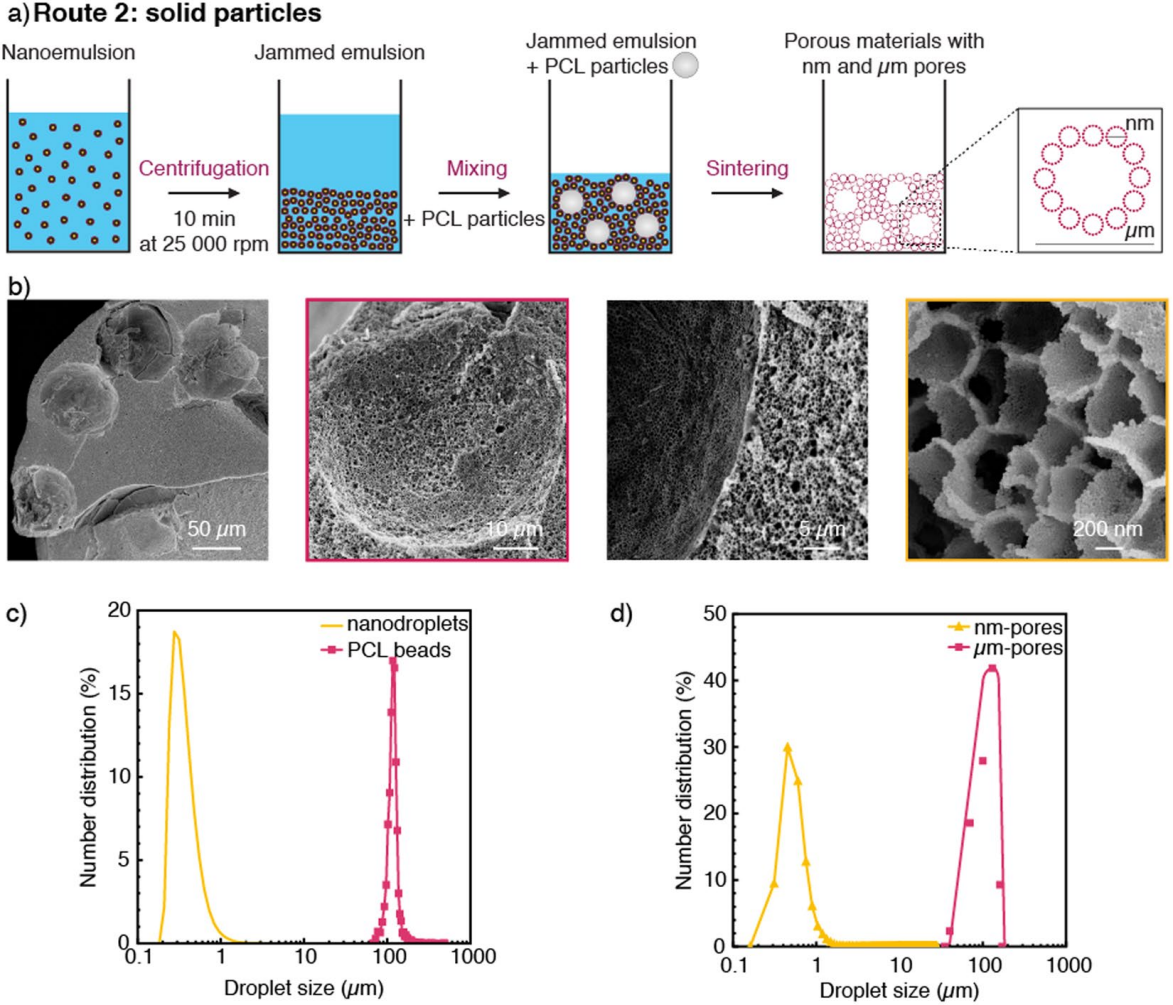
(**a**) Schematics of the processing route used to fabricate hierarchical porous materials from nanoemulsions and PCL particles (Route 2). (**b**) SEM images of the resulting porous structure after sintering. (**c**,**d**) Size distributions of the µm-templates and the nm-sized droplets in (**c**) the wet state and (**d**) after sintering at 850 °C.

The use of polymer particles as sacrificial templates to generate pores at the microscale is an effective alternative route to enhance the fidelity of the templating approach (Route 2, Fig. 4). This is demonstrated using 100 µm monodisperse PCL particles made by microfluidics to form µm-sized pores with narrow size distribution after sintering (Fig. 4a). In this example, the polymer microparticles were added directly to jammed nanoemulsions obtained via centrifugation. Heat treatment of the material at 850 °C enables removal of the liquid phases and polymer particles, resulting in a two-level hierarchical porous structure with well-defined pore sizes. SEM imaging of such structures confirm the strong correlation between the size distribution of pores and that of the original sacrificial template (Fig. 4b). The monodispersity of the particle templates is directly translated into pores, which display a polydispersity index below 0.03 (Fig. 4c,d). This contrasts with the high polydispersity of 0.68 measured for the pores obtained from the large droplet templates (Fig. 3d). Because microfluidic emulsification can lead to monodisperse particles over length scales between 5 µm and 500 µm^34^, the use of such particles as microtemplates in combination with the nanoemulsions described in this study is thus an attractive approach to deliberately design the pores of materials within a large size range.

**Figure 5:**
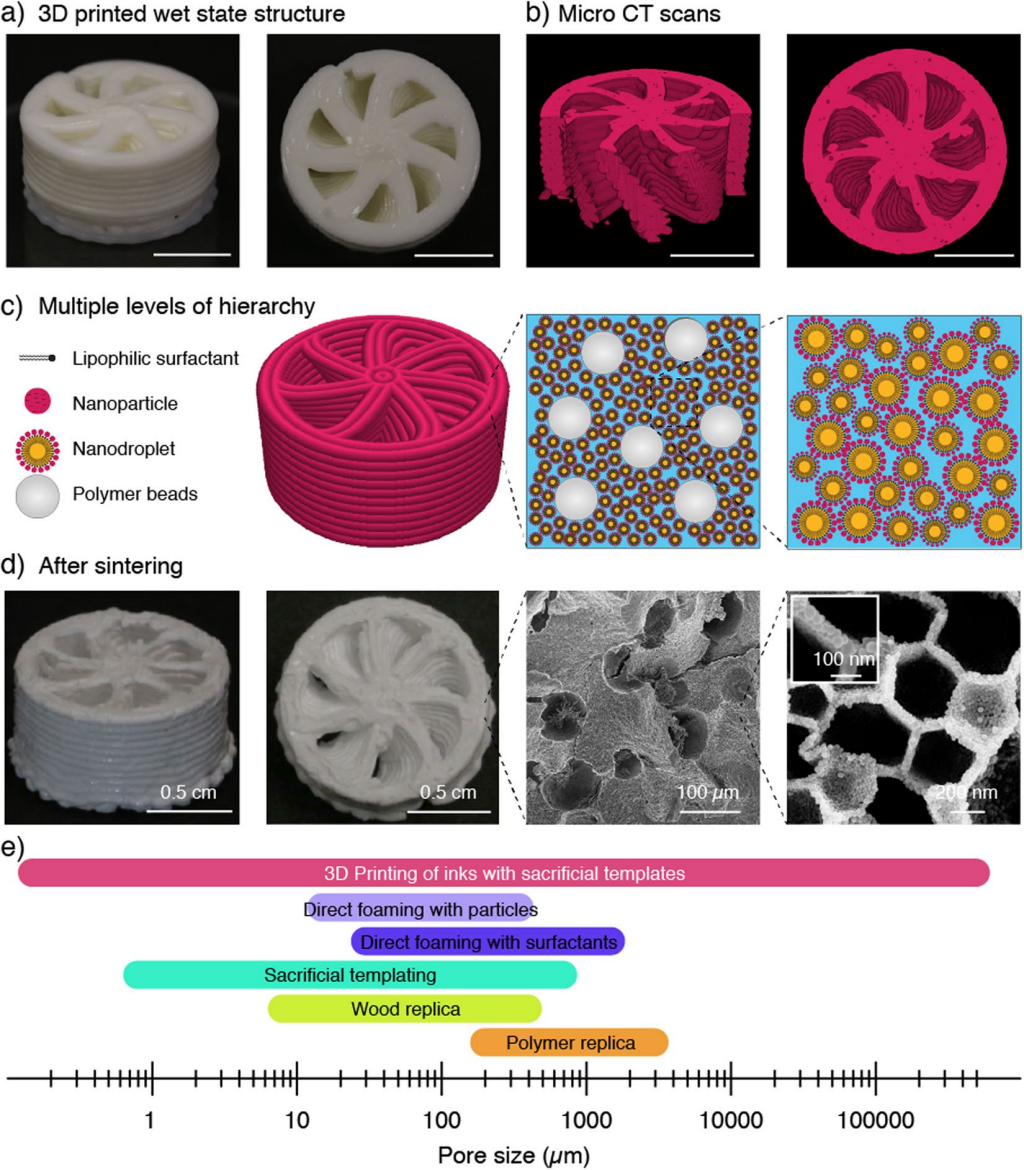
(**a**,**b**) Complex helicoidal structure that was 3D printed using a concentrated nanoemulsion as ink loaded with PCL particles. Images in (**a**) shows the structure in the wet state right after printing, whereas images in (**b**) display reconstructed representations of the printed object from micro-CT scans. Scale bars: 1 cm. (**c**) Schematics of the different building blocks and levels of hierarchy present in the 3D printed structure. (**d**) Photographs and SEM images of the 3D printed structures after drying and sintering. The inset shows that the cell wall of the structure is formed by a single layer of nanoparticles. (**e**) The proposed additive manufacturing technology covers a range of pore sizes that has previously not been accessible by a single processing technique. Scale bars: (**a**) and (**b**), 0.5 cm; (**d**), 0.5 cm, 0.5 cm, 100 µm, 200 nm (left to right) and 100 nm (inset).

3D printing of such dual templating soft materials eventually extends the pore size design space to the millimeter scale and provides a digital means to shape porous architectures into intricate three-dimensional geometries (Fig. 5). Using direct ink writing as the 3D printing technique, the spacing between the extruded filaments can be tailored to control the pore openings at the largest length scale. We demonstrate this possibility by printing water-based inks consisting of jammed nanoemulsions loaded with monodisperse PCL microparticles. Because jammed emulsions exhibit tunable viscoelastic behavior^20^, no further additives are needed to adjust the rheological properties of the ink to meet the flow response required for direct ink writing^35^. In case non-jammed emulsions with lower concentrations of droplets are used as inks, rheological modifiers, such as fumed silica, can be added to tune the flow response of the feedstock material^36^. This ink was eventually printed into a chiral 3D geometry that cannot be easily obtained by conventional manufacturing (Fig. 5a,c). Micro-computed tomography of the printed wet structure reveals more details of the intricate geometry (Fig. 5b). Besides chirality, the printed helicoidal geometry features an internal channel whose walls display the two-level hierarchical porosity arising from the droplet and microparticle templates after sintering at 850 °C (Fig. 5d). Archimedes measurements revealed that the walls of the final chiral structure show 85% and 6% of open and closed porosity, respectively. Grid-like cellular geometries can also be created at the macroscale if the rheology of the ink is tuned with modifiers to prevent sagging of supported filaments^37,38^.

The tortuous nature of the internal channels and the multiscale porous walls make the helicoidal printed geometry potentially interesting for catalytic applications^39,40^, if the ink formulation is adjusted to obtain open porosity after drying and consolidation. Although further studies would be needed to find the optimum hierarchical architecture for this application, the idea is that the internal channels would provide easy accessibility to the high-surface area pores within the walls combined with tunable level of turbulence to promote exchange of reactive species. The fabrication of such structures with heat-resistant inorganic materials enables the utilization of such a catalytic support also at high temperatures. Alternatively, one can envision the utilization of similar 3D printed helicoidal porous structures in thermal management applications. In this case, the pore sizes at coarser length scales would provide high permeability to fluid flow whereas a nanoemulsion containing a non-volatile oil phase would be kept in the structure to allow for thermal storage and release through phase changes. Besides these catalytic and thermal functionalities, such hierarchical porous structures can potentially show improved mechanical efficiency compared to materials featuring the same level of porosity but at one single length scale^18^.

Although several 3D printing and additive manufacturing techniques have been developed to create structures at the nano-, micro- and macroscale, the ability to print porous materials with features spanning over all these multiple length scales in one single fast process is a unique aspect of the proposed technology (Fig. 5e). This is only possible by combining the spatial control offered by the printing technique with the self-assembly nature of the building blocks present in the ink. Eventually, the combination of porosities and pore sizes achieved fall within ranges that have thus far not been accessible by previous processing routes for porous materials^12^.

## Conclusion

Hierarchical porous materials can be fabricated by extrusion-based 3D printing of inks loaded with sacrificial pore templates at the nanometric and micrometric scales. Pore templates may consist of combinations of Pickering nanoemulsions, droplet assemblies and micron-sized polymer particles. The 3D printing process is simple and fast, since the nano- and microporosity are generated from the self-assembly of templating droplets and particles within the ink, as opposed to the slow sequential deposition of material required in other technologies. Because they are susceptible to coalescence during ink preparation, the templating droplets need to be stabilized by particles that will later form the walls of the pores created upon drying and consolidation. The zwitterionic nature of the surfactant used to promote this stabilization mechanism allows for the use of particles with a variety of distinct chemistries. Moreover, the dried printed structure can be consolidated either chemically or via heat treatment, depending on the ink formulation. Combined with the complex shaping capabilities of 3D printing, these features make the process highly tunable and open several new possibilities for the design and digital fabrication of hierarchical porous materials for a variety of applications.

## Experimental Methods

Preparation of particle-stabilized nanoemulsions. Phosphatidylcholine (PC, Lipoid P 100, Lipoid GmbH, Germany) was dissolved at 1 wt_oil_% in corn oil (Sigma-Aldrich, Germany) or in decane (>99%, Acros Organics, Switzerland) at 60 °C. The primary emulsion was prepared using 23.32 wt% of oil and 76.68 wt% of Milli-Q water (18.2 mΩ.cm). Pre-emulsification was achieved with a rotor-stator mixer (Ultra-Turrax, disperser T25 digital, dispersing tool S 25 N – 18 G, IKA, Germany) at 10000 rpm for 5 minutes. Emulsification was performed by passing the oil-water mixture twenty times through a high-pressure homogenizer (HC-5000 equipped with a L30Z microchannel, Microfluidics, United States) at 115 bar. With corn oil, the secondary emulsion contained 86 wt% of the primary emulsion and 14 wt% of an aqueous suspension of silica particles. Two types of commercial silica suspensions were used: Ludox TM50 (50 wt% of 22 nm silica nanoparticles in water, Sigma-Aldrich, Germany) or Ludox CL-P (40 wt% of silica nanoparticles in water, 22 nm, Sigma-Aldrich, Germany). The pH of the Ludox TM50 and Ludox CL-P suspensions was adjusted to pH 5.5 and 4, respectively, using a 1 M HCl solution (Titrisol, Merck, Switzerland). The pH of the primary emulsion was fixed at pH 5.5. The secondary emulsification step was performed by sonication for 5 minutes at a relative intensity of 75% (Sonics, United States). When decane was used as the oil phase, the secondary emulsion was produced by mixing 70 wt% of the primary emulsion with 30 wt% of Ludox TM50 adjusted at pH 5.5. wt_oil_% and wt_O/W_% denotes the weight percentage relative to the total weight of oil and emulsion, respectively.

Zeta potential measurements. The zeta potential of the silica particles was measured using the electroacoustic Colloidal Vibration Current technique in a DT300 equipment at 25 °C (Dispersion Technology, Germany).

Droplet size characterization. The droplet size distribution was evaluated by static light scattering in water (Mastersizer 2000, Malvern Instrument, United Kingdom). A refractive index of 1.47 and an absorption coefficient of 0.0001 were assumed for the oil droplets.

Cryo-scanning electron microscopy. A freeze-fracture technique was used to prepare samples of nanodroplets for imaging in a cryo-SEM^41,42^. Samples were prepared as explained in our previous work^43^.

Fabrication of nanoporous materials. The nanoemulsions were centrifuged for 10 minutes at 25000 rpm (Beckman Avanti J-25 I, GMI, United States). After removal of the supernatant, nanoporous materials are obtained by drying the emulsion at room temperature or sintering at 850 °C in a furnace (LE 6/11/P300, Nabertherm, Germany).

Fabrication of hierarchical porous materials. Route 1: Oil-in-water (O/W) emulsions containing microdroplets coated with nanodroplets were obtained by mixing decane containing 1 wt_oil_% of PC with the nanoemulsion previously described in a 4:6 weight ratio. In this case, the nanoemulsion was prepared with decane and 15 wt_O/W_% of silica. Such mixture was sonicated for 5 minutes at a relative intensity of 75% (Sonics, United States). A hierarchical porous material was obtained after centrifugation of the emulsion for 3 minutes at 2500 rpm (Centrifuge 5417 R, Eppendorf, Switzerland) and drying at room temperature. Route 2: Polycaprolactone (PCL, M_w_ ≈ 14000 g.mol^−1^, Sigma Aldrich) beads were produced by microfluidic step emulsification as reported in the literature^44^. Briefly, 5 wt% of PCL was dissolved in dichloromethane (DCM, Acros Organics, Switzerland) and emulsified in water with 2 wt% poly(vinyl alcohol) (PVA, M_w_ = 31000–50000 g.mol^−1^) as the surfactant. The channel design of the microfluidic glass chip and the PCL concentration in DCM were adjusted to generate monodisperse beads with an average size of 100 µm. The emulsions were immersed in pure water for a week until complete evaporation of DCM and the obtained beads were used afterward as PCL microtemplates. For the preparation of the hierarchical materials, the PCL beads were centrifuged for 2 min at 2000 rpm (Hermle Z306, Hermle Labortechnik GmbH, Germany) before being added to a centrifuged nanoemulsion (10 min, 25000 rpm) at a concentration varying from 10 to 20 wt%. The hierarchical porous material was obtained after sintering at 850 °C.

3D printing by Direct Ink Writing. An helicoidal-shaped porous structure was printed using a pressure controlled direct-ink-writing system (3DDiscovery, RegenHu, Switzerland). A nanoemulsion containing PCL particles was used as ink. Such jammed emulsion showed the rheological properties required to produce distortion-free 3D structures^35,37,38^. The filament diameter was set to 0.58 mm. After the printing process, samples were dried and sintered at 850 °C.

Characterization. SEM images were taken with a LEO 1530 instrument (Zeiss GmbH, Germany). Image analysis of the droplet and pore size was conducted using the software Fiji. Micro-CT scans were obtained using a µCT 100 (10 µm voxel size, 45 kVp, 88 uA, 200 ms integration time, SCANCO Medical, Brüttisellen, Switzerland).

## Electronic supplementary material

Supplementary Information
