# Supplementary material for: 3D printing of sacrificial templates into hierarchical porous materials
Source: Sci Rep. 2019 Jan 23;9:409. doi: 10.1038/s41598-018-36789-z (PMC6344549; doi:10.1038/s41598-018-36789-z)
Supplement: Supplementary file 1 — Supplementary Information [file 41598_2018_36789_MOESM1_ESM.docx]

Supplementary Information

3D printing of sacrificial templates into hierarchical porous materials

Lauriane Alison, Stefano Menasce, Florian Bouville, Elena Tervoort, Iacopo Mattich, Alessandro Ofner, André R. Studart*


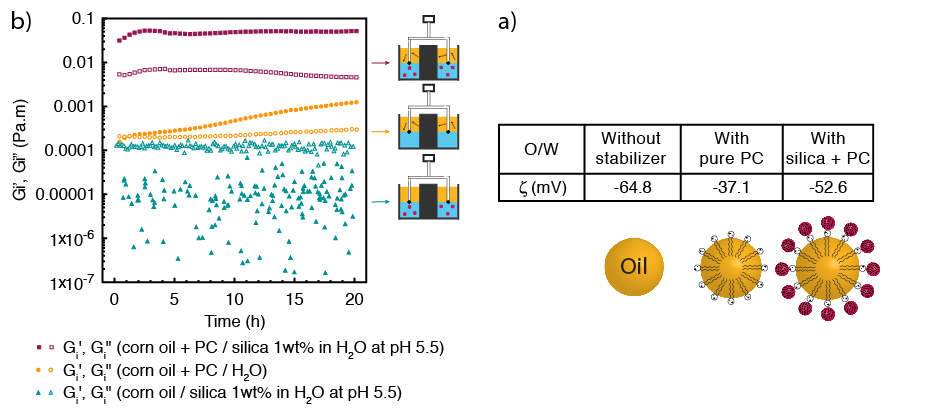


**Figure S1.** (a) Interfacial rheology measurements revealing the formation of a viscoelastic layer at the oil-water interface when PC and silica nanoparticles adsorb simultaneously at the oil-water interface. Time sweep performed at an amplitude strain γ = 0.3% and an angular frequency ω = 1 rad.s^-1^. Silica suspensions were prepared with 1 wt_susp_% SiO_2_ at pH 5.5. When water was used alone as a lower phase, the pH was not adjusted. (b) Zeta potential values of corn oil droplets demonstrating that phosphatidylcholine (PC) molecules with net positive charge reduce the negative surface of oil droplets upon adsorption. Despite the net negative zeta potential of the PC-coated droplets, other interactions possibly involving the positive charges in the zwitterionic PC molecules must occur to enable the adsorption of silica negative nanoparticles at the oil-water interface. This is confirmed by the more negative zeta potential of droplets containing both silica and PC as compared to only PC. Measurements were performed at pH 5.5.


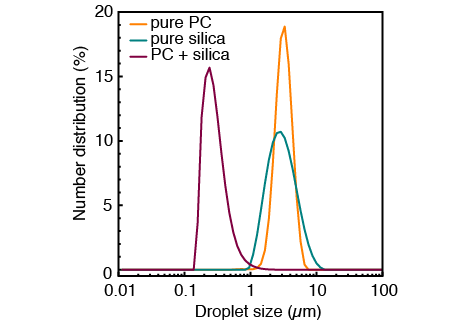


**Figure S2.** Droplet size distribution of emulsions prepared with different stabilizers showing the size reduction enabled by the co-stabilization mechanism using PC and silica nanoparticles. Larger droplets are obtained if the stabilizer diffuses too slowly to the oil-water interface (pure silica) or if the droplets are not sufficiently stable against coalescence and coarsening (PC). Instead, the combination of fast diffusing surface active molecules (PC) and strong interfacially adsorbed particles (silica) leads to smaller stable droplet sizes (PC + silica). The oil-in-water emulsion was prepared by using a 7 wt_O/W_% silica at a pH of 5.5 or/and corn oil containing 1 wt_oil_% of PC. The oil-to-water weight ratio of the emulsion was fixed at 2:8.


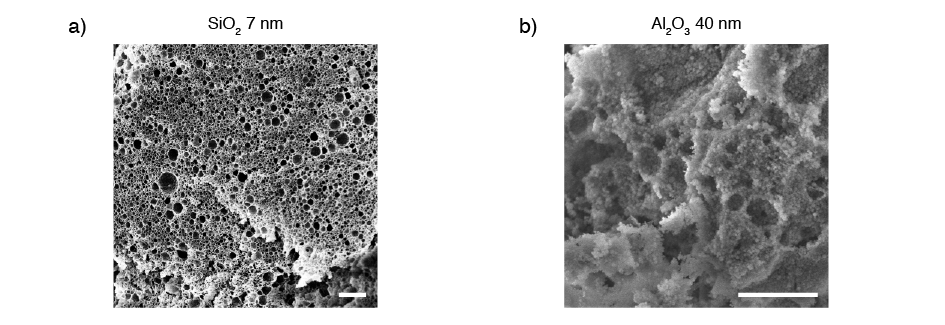


**Figure S3.** SEM images of centrifuged nanoemulsions prepared with (a) smaller silica nanoparticles and (b) alumina particles. Scale bars: 2 µm.


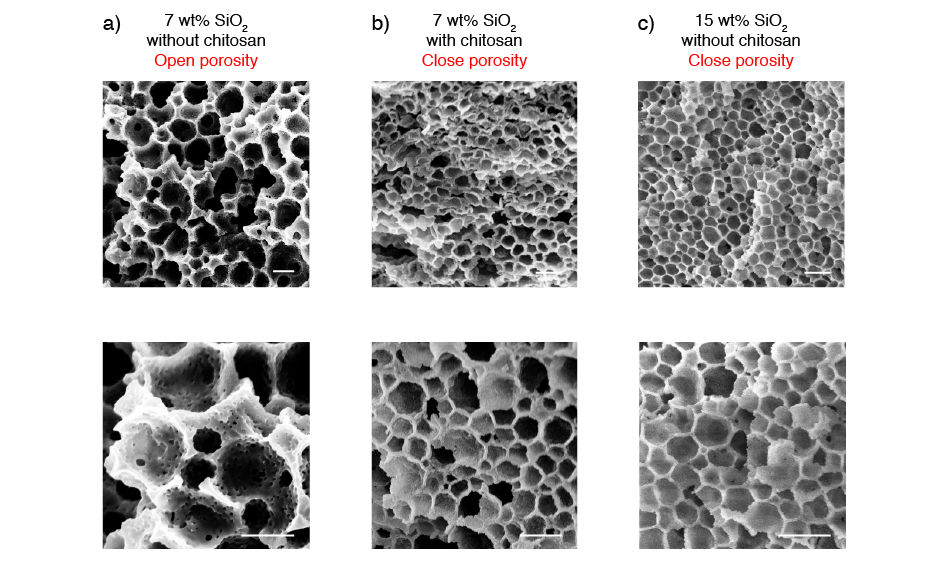


**Figure S4.** SEM images of porous structures obtained from decane-in-water nanoemulsions stabilized by PC and silica nanoparticles at a pH of 5.5. The oil phase contains 1 wt_oil_% of PC. Changing the compositions of the nanoemulsions allow to tailor the pore characteristics. Oil-in-water emulsions were prepared from (a) 7 wt_O/W_% silica suspension, (b) 7 wt_O/W_% silica suspension modified with 1 wt_SiO2_% of chitosan and (c) 15 wt_O/W_% silica suspension. The oil-to-water weight ratio of the emulsion was fixed at 2:8. Scale bars: A, B, C, 1 µm.


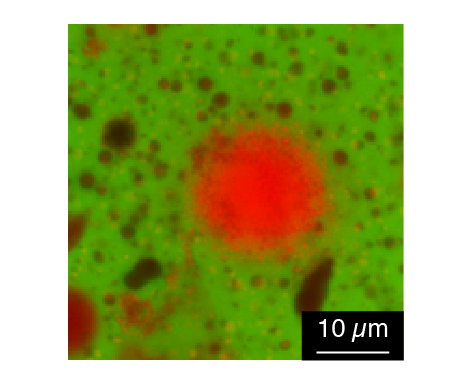


**Figure S5.** Confocal microscopy image of hierarchical oil-in-water droplet assembly. In this assembly, the large oil droplets (in black) are covered by much smaller oil nanodroplets (in red). Nanodroplets were prepared with a red-fluorescent dye to facilitate their visualization. The droplet assembly was prepared using 1 wt_oil_% of PC in corn oil, whereas the initial nanoemulsion contained 15 wt_O/W_% of silica. The oil-to-water ratio was 4:6. Corn oil was used here as an example. Similar structures are expected if decane is utilized as oil phase.


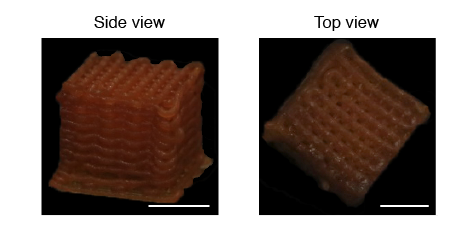


**Figure S6.** Photographs of the 3D-printed cubic lattice after consolidation with 5 wt% chitosan and 2.5 wt% glutaraldehyde. Scale bars: 5 mm.

**Experimental section**

Fabrication of nanoporous materials with other particles:

Ludox SM30 (30 wt% of silica nanoparticles in water, 7 nm, Sigma-Aldrich, Germany) and aluminium oxide (99.5%, NanoArc, 40 nm, Alfa Aesar, Germany) were used to replace the Ludox TM50 particles utilized in the experiments shown in the main text. The particle concentration was fixed at either 7 wtO/W% or 15 wtO/W% with respect to the total emulsion. The nanoemulsion containing chitosan was prepared with 7 wtO:W% of chitosan-modified silica particles with respect to the total emulsion. The chitosan modification was conducted with 1wtSiO2% of chitosan, as explained in our previous work.1 wtoil%, wtO/W% wtsusp% and wtSiO2% denotes the weight percentage relative to the total weight of oil, emulsion, aqueous suspension and silica nanoparticles.

Zeta potential measurements:

The zeta potential values of the oil droplets were measured using the electrophoretic technique in a Zetasizer Nano ZS at 25°C (Malvern, United Kingdom). The nanoemulsions were diluted to an oil-water volume ratio of 1:100 prior to the measurements.

Rheological measurements:

Interfacial rheology measurements were performed at 25°C with a rheometer (HR 3 Discovery Hybrid Rheometer, TA instruments, United States) equipped with a double-wall ring placed at the oil-water interface.^2^ Time sweeps were performed at an amplitude strain γ = 0.3% and an angular frequency ω = 1 rad.s^-1^ for 20 h.

Consolidation of the porous materials without sintering:

Hierarchical porous materials prepared with decane can be solidified via chemical crosslinking if a binder is added to the continuous aqueous phase. Chitosan powder (chitosan oligosaccharide, M_w_ ≤3 kDa, Haide Bei, China) was added at 5 wt% to the liquid nanoemulsion and stirred prior centrifugation until the obtaining of a homogeneous suspension. The centrifuged paste was used as ink for 3D printing. The resulting structure was then exposed to a 2.5 wt% of glutaraldehyde (50 wt% in water, Sigma-Aldrich, Germany) solution vapor in a humidity chamber (HCP108, Memmert, Germany) for 3 days at 30°C and 95% RH.

**References**

1. Alison, L.; Rühs, P. A.; Tervoort, E.; Teleki, A.; Zanini, M.; Isa, L.; Studart, A. R., Pickering and Network Stabilization of Biocompatible Emulsions Using Chitosan-Modified Silica Nanoparticles. *Langmuir* **2016,** *32* (50), 13446-13457.

2. Vandebril, S.; Franck, A.; Fuller, G. G.; Moldenaers, P.; Vermant, J., A double wall-ring geometry for interfacial shear rheometry. *Rheol Acta* **2010,** *49* (2), 131-144.
